# CCR2 knockout ameliorates obesity-induced kidney injury through inhibiting oxidative stress and ER stress

**DOI:** 10.1371/journal.pone.0222352

**Published:** 2019-09-09

**Authors:** Seung Joo Lee, Jeong Suk Kang, Hong Min Kim, Eun Soo Lee, Ji-Hye Lee, Choon Hee Chung, Eun Young Lee

**Affiliations:** 1 Department of Internal Medicine, Soonchunhyang University Cheonan Hospital, Cheonan, Korea; 2 Institute of Tissue Regeneration, College of Medicine, Soonchunhyang University, Cheonan, Korea; 3 Department of Internal Medicine, Yonsei University Wonju College of Medicine, Wonju, Korea; 4 Department of Pathology, Soonchunhyang University Cheonan Hospital, Cheonan, Korea; University Medical Center Utrecht, NETHERLANDS

## Abstract

CCL2/CCR2 signaling is believed to play an important role in kidney diseases. Several studies have demonstrated that blocking of CCR2 has a therapeutic effect on kidney diseases. However, the effects of CCR2 knockout on obesity-induced kidney injury remain unclear. We investigated the therapeutic effects and the mechanism of CCL2/CCR2 signaling in obesity-induced kidney injury. We used *C57BL/6*-CCR2 wild type and *C57BL/6*-CCR2 knockout mice: Regular diet wild type (RD WT), RD CCR2 knockout (RD KO), High-fat diet WT (HFD WT), HFD CCR2 KO (HFD KO). Body weight of WT mice was significantly increased after HFD. However, the body weight of HFD KO mice was not decreased compared to HFD WT mice. Food intake and calorie showed no significant differences between HFD WT and HFD KO mice. Glucose, insulin, total cholesterol, and triglycerides levels increased in HFD WT mice were decreased in HFD KO mice. Insulin resistance, increased insulin secretion, and lipid accumulation showed in HFD WT mice were improved in HFD KO mice. Increased desmin expression, macrophage infiltration, and TNF-α in HFD mice were reduced in HFD KO mice. HFD-induced albuminuria, glomerular hypertrophy, glomerular basement membrane thickening, and podocyte effacement were restored by CCR2 depletion. HFD-induced elevated expressions of xBP1, Bip, and Nox4 at RNA and protein levels were significantly decreased in HFD KO. Therefore, blockade of CCL2/CCR2 signaling by CCR2 depletion might ameliorate obesity-induced albuminuria through blocking oxidative stress, ER stress, and lipid accumulation.

## Introduction

Obesity is a major public health problem in both developed countries and developing countries [1]. World Health Organization has predicted that the number of obese people in the world will increase from at least 400 million in 2005 to 700 million in 2015 [1]. Obesity contributes to complex systemic inflammatory state associated with medically important complications such as atherosclerosis, hepatic steatosis, and insulin resistance [2–4]. It is well known that obesity can induce cardiovascular and renal diseases through several mechanisms of hypertension, hyperglycemia, inflammation, and dyslipidemia [5]. In kidney, obesity induces many other risk factors such as hyperinsulinemia, hyperlipidemia, impaired renin angiotensin-aldosterone activity, oxidative stress, and insulin resistance [6]. Reduced insulin sensitivity is more likely to lead to chronic kidney disease than other metabolic complications in the kidney [7].

Inflammatory cytokines including C-C chemokine ligand 2 (CCL2), TNF-α, and interleukin-6 are increased in obesity [8–10]. It has been implicated that obesity can upregulate circulating CCL2 and TNF-α, the cause of obesity-associated insulin resistance and the development of type 2 diabetes [8, 11–14]. CCL2, also known as monocyte chemoattractant protein-1, is a C-C chemokine family. It initiates inflammation by binding to C-C chemokine receptor 2 (CCR2) [15]. An upregulation of CCR2 occurs before macrophage infiltration in human diabetic nephropathy [16].

In obesity, both fatty acids oxidation and reactive oxygen species (ROS) production in mitochondria are increased [17, 18]. The enzymatic activity of NADPH oxidase (Nox) plays a major role of ROS generation in various signaling pathways [19, 20]. Nox4, one of Nox isoforms, is most abundantly expressed in the endoplasmic reticulum in some cells [21]. Originally identified in the renal cortex, Nox4 is abundant in the distal tubules, collecting ducts, renal papilla epithelium, and mesangial cells of kidney [22, 23]. Inflammation and ER stress are increased through interactions between CCL2 and CCR2 [24].

In human and experimental models, CCL2 contributes to a variety of renal diseases, including progressive renal damage such as chronic rejection of renal transplantation, lupus nephritis, lgA nephropathy, and diabetic nephropathy [25–28]. In experimental diabetes, CCL2 is overexpressed within the glomeruli [29, 30]. Several studies have reported that the development of diabetic nephropathy could be blocked in type 2 diabetic mice by using CCR2 inhibitors such as RS504393 and RO523444 [31, 32]. Our previous study has demonstrated that the function and structure of kidney could be improved by CCR2 inhibitor treatment in type 2 diabetic mice model [33]. Most kidney studies on CCL2/CCR2 have reported that kidney function can be recovered by treatment with CCR2 inhibitor in type 2 diabetes through reducing albuminuria and changing glomerular structure *et al* [31–33]. Tarabra *et al*. have demonstrated that the expression levels of nephrin, synaptopodin, and zonula occludens-1 in CCL2 knockout mice are recovered when compared to those in control diabetic mice [34]. However, the effect of CCR2 KO on obesity-induced kidney injury remains unclear.

In this study, we investigated the therapeutic effect of CCR2 KO on obesity-induced kidney damage including renal function and glomerular structure.

## Materials and methods

### Ethics statement

All experiments were performed under the approval of the Institutional Animal Care and Use Committee of Yonsei University at the Wonju Campus (YWC-140320-1). Daily inspections were performed to minimize animal suffering and mice with signs of disease or discomfort were euthanized by CO2 and cervical dislocation. Surgical tissue isolations were performed as terminal procedures under anesthesia as described below.

### Animal model

Male *C57BL/6*-CCR2 wild type and *C57BL/6*-CCR2 knockout mice at 6 weeks old were purchased from Jackson Laboratory (Bar Harbor, ME, USA). Mice from 8 to 18 weeks of age were used in this study. Mice were divided into four groups: Regular diet wild type (RD WT; *n* = 9), RD CCR2 knockout (RD KO; *n* = 9), High-fat diet WT (HFD WT; *n* = 9), HFD CCR2 KO (HFD KO; *n* = 9). Mice were fed either normal chow or high-fat diet chow (Research Diets D12492, 5.24kcal, protein 20%, carbohydrate 20%, fat 60%; Research Diets, New Brunswick, NJ, USA) for 10 weeks, starting from 8 weeks of age. Body weight, food intake, and calories were measured every week from 8 to 18 weeks of age. The animals at 18 weeks of age were anesthetized with Zoletil (Virvac Laboratories, Carros, France) and xylazine hydrochloride (Rompun TS, Bayer AG, Leverkusen, Germany) at dose rates of 4.0 mg/kg and 2.2 mg/kg body weight by intraperitoneal injection respectively. Serum was collected and weights of the kidney, liver, pancreas, muscle, and white adipose tissue (WAT) were measured after tissue collection. All samples were stored at -80°C for further studies. Homeostasis model assessment (HOMA-IR) and homeostasis model assessment of β-cell function (HOMA-β) were calculated using the following formula to confirm insulin resistance and secretion: HOMA-IR = fasting plasma glucose (mmol/L) × fasting serum insulin (mU/L) ÷ 22.5; HOMA-β = 20 × fasting serum insulin (mU/L) ÷ fasting plasma glucose (mg/dL)– 3.5.

### Podocyte culture and treatment

Conditionally immortalized mouse podocytes were kindly provided by Dr. Peter Mundel (Harvard Medical School, Boston, MA, USA). Generally, podocytes were cultured at 33°C under permissive conditions in DMEM supplemented with 10% FBS and 10 U/ml mouse recombinant interferon-γ (Sigma-Aldrich, St. Louis, MO, USA) to enhance the expression of a thermosensitive T antigen. To induce differentiation, podocytes were grown under nonpermissive conditions at 37°C without interferon-γ for 14 days. Before the application of high glucose (HG), the cells were maintained under serum-deprived conditions for 24 h and harvested for the next assay.

### Measurements of glucose tolerance test

Glucose tolerance test was measured at 8 weeks after starting HFD. After 8 hours of fasting, glucose (1.0 g/kg) was administered by intraperitoneal injection. Blood sample was obtained from tail vein and measured by Auto-Chek (Diatech Korea, Seoul, Korea).

### Measurement in serum

After mice were sacrificed after 8 hours of fasting period, blood samples were collected by cardiac puncture. Serum concentrations of glucose, total cholesterol, triglycerides, Glutamic-oxaloacetic transaminase (GOT), and glutamic-pyruvic transaminase (GPT) were measured using commercially available enzymatic assay kits (Asan Pharmacology, Seoul, Korea). Serum insulin level was analyzed with an ultrasensitive mouse insulin ELISA kit (Shibayagi, Gunma, Japan).

### Measurements of albuminuria

To measure albuminuria, 24 hours urine was collected using metabolic cages. Urinary albumin (Exocell NephratII; Exocell Inc., Philadelphia, PA, USA) and creatinine (The Creatinine Companion; Exocell Inc.) levels were measured using ELISA kit.

### Histological assessment of kidney, liver, and WAT

For immunohistochemical staining, paraffin-embedded kidney samples were sliced into 4-μm-thick sections. Deparaffinized samples were incubated with anti-desmin (1:100, Abcam, Cambridge, UK) or anti-F4/80 (1:100, Santa Cruz, Texas, USA) at 4°C overnight. After washing three times, samples were incubated with anti-rabbit IgG (Thermo Scientific, MA, USA) secondary antibody at room temperature for 30 minutes. Samples were counterstained with hematoxylin and dehydrated with xylene and ethanol. Histological changes were observed under a microscope (Olympus, Tokyo, Japan). In WAT and kidney hematoxylin and eosin (H&E) staining, adiposity size and glomerular volume were calculated using an image processing and analysis software, Image J (National Institutes of Health, Bethesda, MD, USA). Desmin and F4/80 expression score were calculated semiquantitatively. The level of staining was graded as follow: 0, 0%; 1+, 1–25%; 2+, 26–50%; 3+, 51%-75%; 4, 76%-100%.

### Immunofluorescence

Cultured podocytes grown on collagen-coated coverslips for 14 days were fixed with 4% paraformaldehyde, permeabilized with 0.25% Triton X-100, blocked with 1% BSA, and immunolabeled with FITC-phalloidin (Sigma-Aldrich) and Paxillin (Invitrogen, Carlsbad, CA, USA) for 1 h at room temperature. The images were collected using an LSM 510 META laser-scanning confocal microscope (Carl Zeiss Microimaging, Thornwood, NY, USA).

### Transmission electron microscopy of kidney

For transmission electron microscopic (TEM) observations, samples were fixed in 2.5% glutaraldehyde for 2 hours at 4°C, washed with 0.1M phosphate buffer at pH 7.4, and then fixed in 1% osmium tetroxide for 90 minutes. Samples were dehydrated with a graded series of ethanol, exchanged in propylene oxide, and embedded with mixture of Epon. Electron micrographs of each sample were taken at ×30k. Slit pores density was measured with image analysis system (GmbH, sis, Minster, Germany) and divided by glomerular basement membrane (GBM) length (10-μm) to obtain linear density. The GBM thickness was counted from measurements at three different cross-sectioning sites.

### RNA extraction and quantitative real-time PCR

Total RNA was extracted from kidney cortex using TRIzol (Ambion, CA, USA) according to the manufacturer's instruction. cDNA was synthesized from 1.0 μg total RNA using ReverTra Ace qPCR RT kit (TOYOBO, Osaka, Japan). Quantitative real-time PCR was performed using SYBR Green PCR master mix (TOYOBO, Osaka, Japan) on a CFX connected Real-time system (BIO RAD, California, USA). The following primer sets were used: *ms xBP1* spliced, S: 5’-TGA GTC CGC AGC AGG TG-3’ and AS: 5’-GCA GAC TCT GGG GAA GGA C-3’; *ms xBP1* unspliced, S: 5’-AAG AAC ACG CTT GGG AAT GG-3’ and AS: 5’-CAT AGT CTG AGT GCT GCG GA-3’; *ms Nox4*, S: 5’-AAA AAT ATC ACA CAC TGA ATT CGA GAC T-3’ and AS: 5’-TGG GTC CAC AGC AGA AAA CTC-3’; *ms β-actin*, S: 5’-GGA CTC CTA TGT GGG T-3’ and AS: 5’-CTT CTC CAT GTC GTC CCA GT-3’. Gene expression was calculated using the 2^-ΔΔCt^ method by normalizing with β-actin gene expression in all experiments.

### Western blotting

Cultured podocytes and kidneys were homogenized in PRO-PREP^TM^ protein extraction solution (iNtRON Biotechnology, Seoul, Korea) containing protease inhibitor cocktail (Roche Diagnostics GmbH, Mannheim, Germany). All samples were quantified using the Bradford assay (Bio-Rad, Hercules, CA, USA) with BSA as a standard, and an equal amount of each lysate was examined by SDS-PAGE. The separated proteins were transferred to a polyvinylidene fluoride membrane (Millipore, Billerica, MA, USA). The membrane was blocked with 5% nonfat dry milk followed by primary antibody incubation at 4°C overnight. Primary antibodies for xBP1 (Santa Cruz Biotechnology), Bip (Santa Cruz Biotechnology), nephrin (Progen Biotechnik, Heidelberg, Germany), Nox4 (Santa Cruz Biotechnology), TNF-α (Cell Signaling, Danver, MA, USA), Collagen IV (SouthernBiotech, Birmingham, AL, USA) were prepared in 0.1% Tris-buffered saline containing Tween-20 and 1% milk at an appropriate dilution. Subsequently, the membranes were washed with phosphate-buffered saline-Tween solution followed by incubation with horseradish peroxidase-conjugated secondary antibody. The bands were visualized with a ChemiDoc^TM^ XRS+ (Bio-Rad, Hercules, CA, USA) imaging system using a Luminata Forte enhanced chemiluminescence solution (Millipore).

### Statistical analysis

Data are presented as mean ± standard error of the mean (SEM). A *t*-test was used to compare results between two groups. A *P*-value < 0.05 was considered as statistically significant.

## Results

### Effects of CCR2 depletion on clinical characteristics of mice

**Fig 1:**
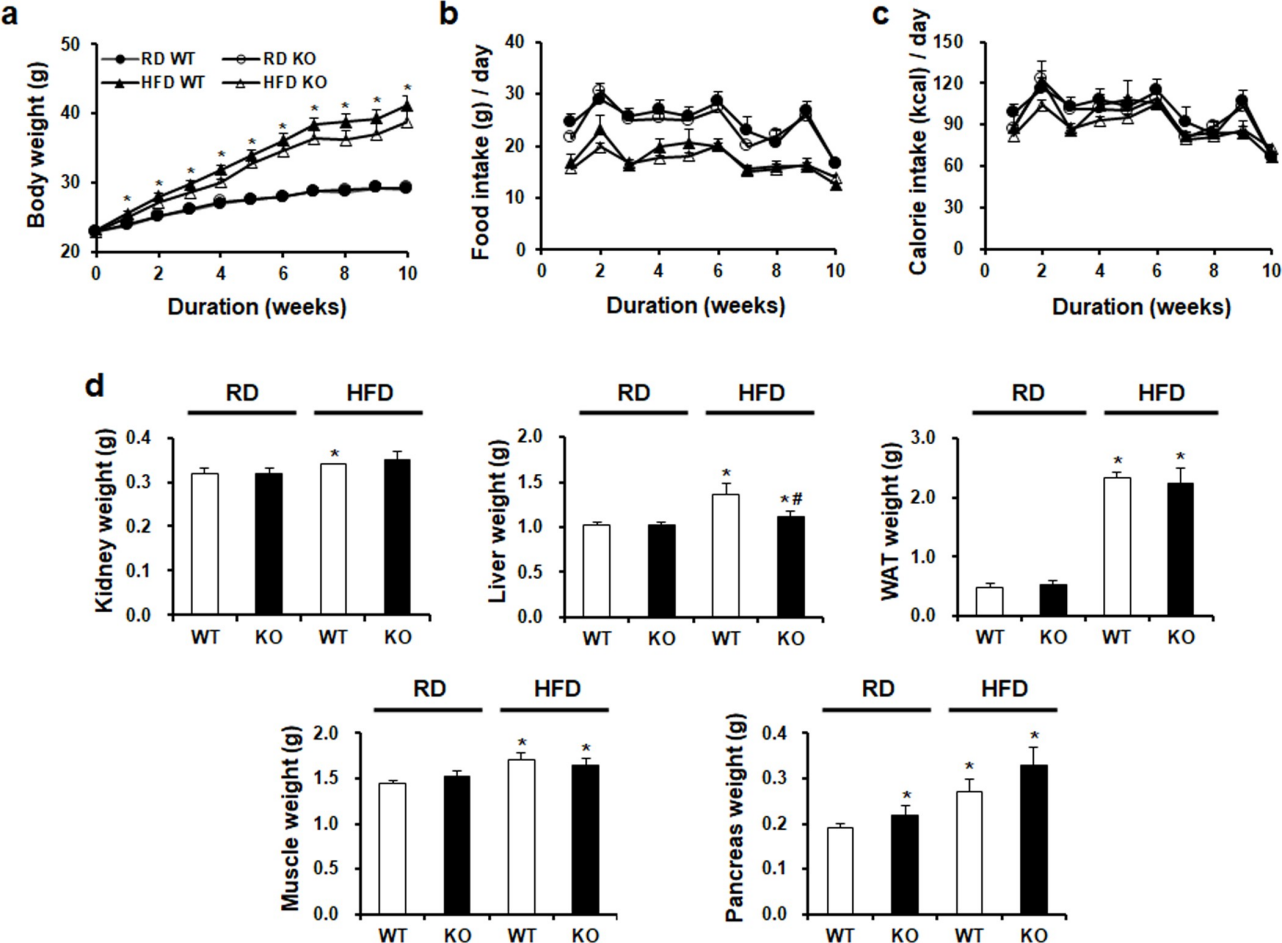
Changes in body weight, food intake, calorie intake, and tissue weight in the four experimental groups. WT and CCR2 KO mice were fed HFD or RD for 10 weeks. (a) Body weight changes over time. (b) Food intake changes over time. (c) Calorie showed no significant difference among the four groups. (d) Weights of kidney, liver, WAT, pancreas, and skeletal muscle were increased by HFD. However, only liver weight was significantly decreased by CCR2 depletion in HFD KO. RD WT, Regular diet wild type; RD KO, Regular diet CCR2 knockout; HFD WT, High-fat diet wild type; HFD KO, High-fat diet CCR2 knockout; WAT, White adiposity tissue. *P < 0.05 compared to that in RD WT; #P < 0.05 compared to that in HFD WT.

60% HFD induced significantly weight gain in *C57BL/6J* WT mice. However, HFD-induced increase of body weights was not decreased by CCR2 depletion in HFD KO mice (Fig 1A). In the case of food intake, RD WT mice had higher food intake than HFD WT mice (Fig 1B). Food intake of HFD KO mice was not significantly different from that of HFD WT mice. Calorie intake showed no significant difference between HFD KO mice and HFD WT mice (Fig 1C). Weights of kidney, liver, white adipose tissue (WAT), pancreas, and muscle in the HFD groups were significantly upregulated. However, only liver weight on HFD KO mice was significantly decreased by CCR2 depletion compared to HFD WT (Fig 1D).

### CCR2 knockout improves blood glucose levels

**Fig 2:**
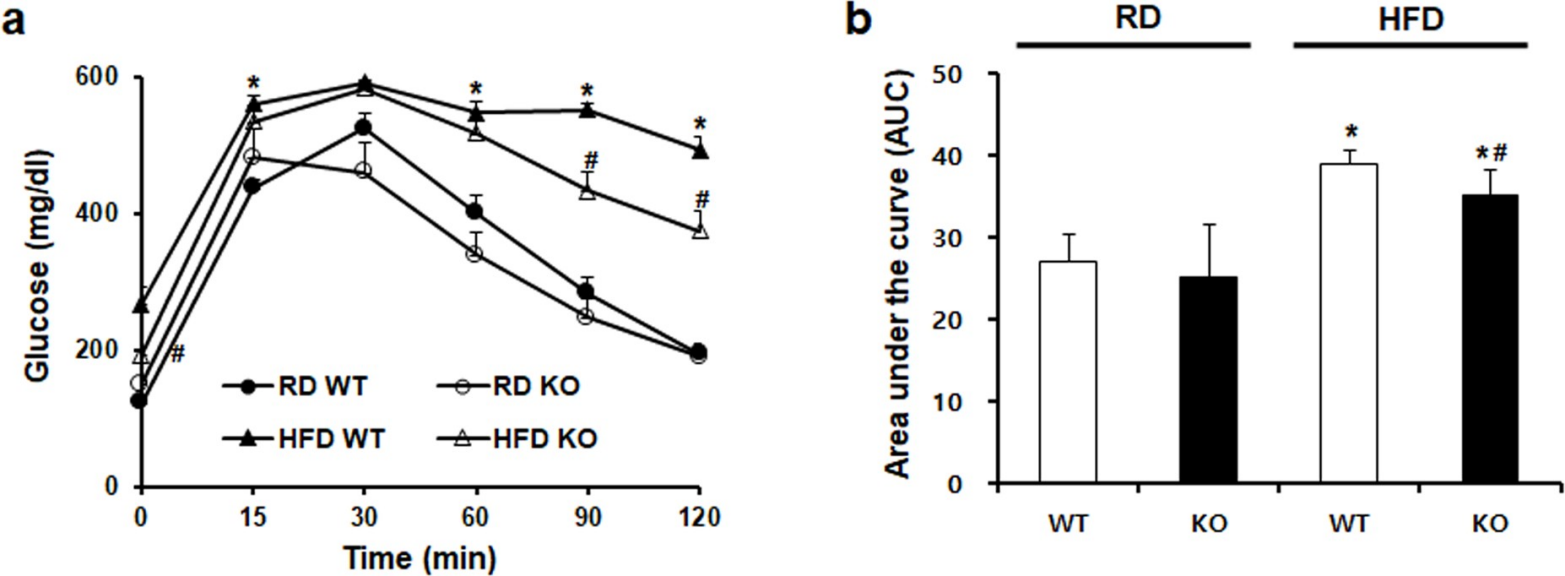
The effect CCR2 depletion on glucose tolerance. (a) Glucose tolerance test (GTT) was performed after 8 weeks of HFD feeding and (b) calculated serum glucose area under the curve by intraperitoneal injection (1.0 g/kg). Increased glucose levels in HFD WT were remarkably decreased in HFD KO mice. RD WT, Regular diet wild type; RD KO, Regular diet CCR2 knockout; HFD WT, High-fat diet wild type; HFD KO, High-fat diet CCR2 knockout. *P < 0.05 compared to that in RD WT; #P < 0.05 compared to that in HFD WT.

As shown in Fig 2A, blood glucose levels after glucose challenge were significantly increased in HFD WT mice. CCR2-depeleted HFD KO mice showed markedly improved blood glucose levels at 90 min and 120 min compared to HFD WT mice. Further in calculated serum glucose area in the curve, HFD-induced glucose increase was also significantly decreased by CCR2 depletion in HFD KO mice (Fig 2B).

### CCR2 knockout improves insulin resistance and secretion

**Fig 3:**
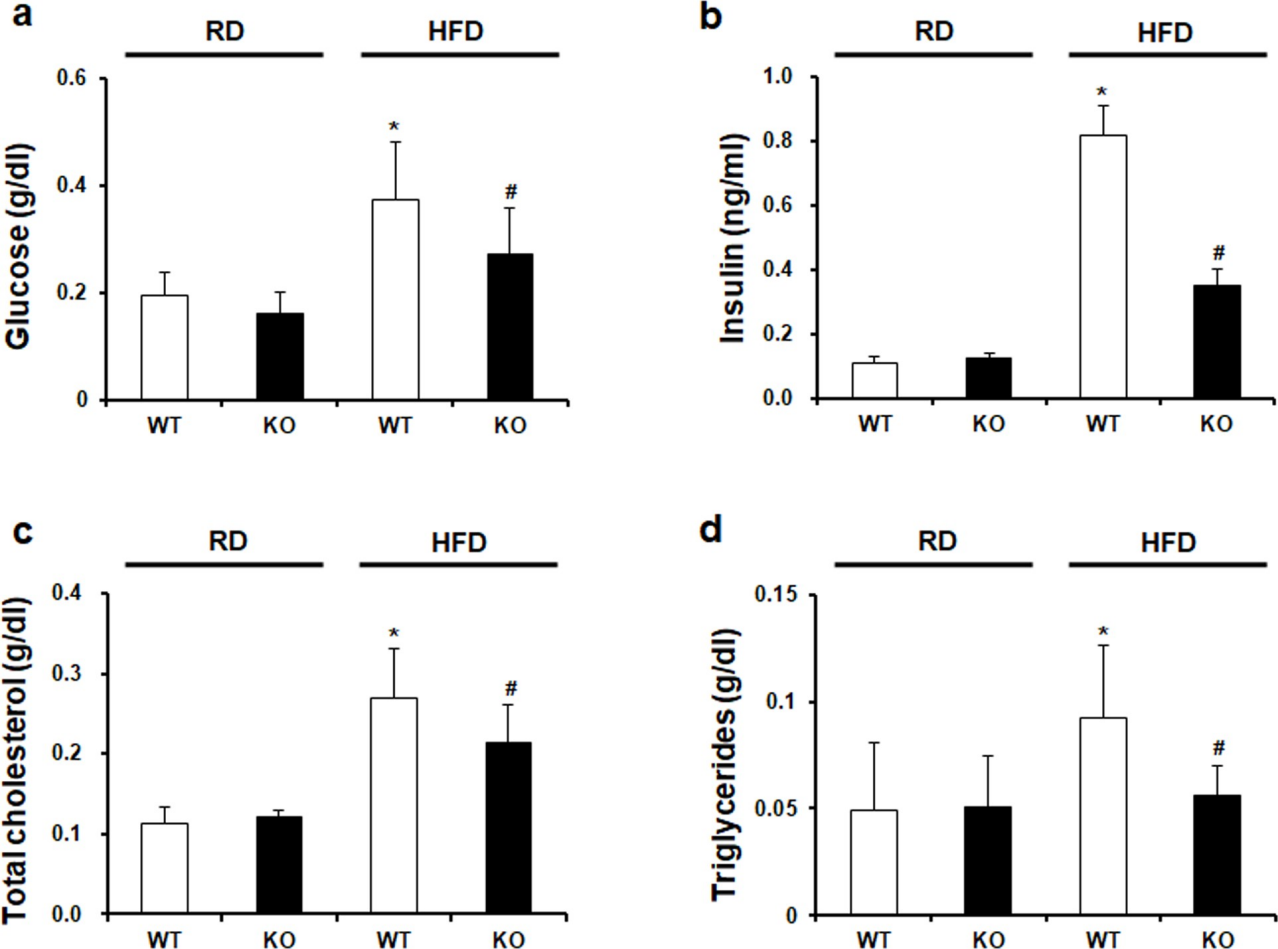
Biochemical characteristics in experimental animals. (a) Glucose, (b) insulin, (c) total cholesterol, and (d) triglycerides in mouse serum samples were measured by ELISA kit. (a-d) Significantly increased glucose, insulin, total cholesterol, and triglycerides in HFD WT were markedly decreased in HFD KO. RD WT, Regular diet wild type; RD KO, Regular diet CCR2 knockout; HFD WT, High-fat diet wild type; HFD KO, High-fat diet CCR2 knockout. *P < 0.05 compared to that in RD WT; #P < 0.05 compared to that in HFD WT.

We analyzed serum glucose and insulin levels. As shown in Fig 3A and 3B, HFD-induced upregulation of glucose and insulin was downregulated by CCR2 depletion in HFD KO mice. HOMA-IR, insulin resistance index was significantly increased in HFD WT mice. However, it was effectively attenuated in HFD KO mice. HOMA-β, an indicator of insulin secretion from pancreatic β-cells, was also significantly increased in HFD WT mice. In HFD KO mice, HOMA-β level was significantly decreased by CCR2 depletion compared to HFD WT mice (Table 1).

**Table 1:** The effect of CCR2 depletion on insulin resistance and secretion.

Parameters	RD WT	RD KO	HFD WT	HFD KO
**HOMA-IR**	0.02 ± 0.00	0.02 ± 0.00	0.29 ± 0.05*	0.10 ± 0.02*^#^
**HOMA-β**	0.12 ± 0.02	0.20 ± 0.04*	0.40 ± 0.07*	0.24 ± 0.02*^#^

HOMA-IR and HOMA-β are insulin resistance and insulin secretion index, respectively. Significant increases in insulin resistance and secretion in HFD WT were dramatically decreased in HFD KO. HOMA index was calculated using the following formula: HOMA-IR = fasting plasma glucose (mmol/L) × fasting serum insulin (mU/L) ÷ 22.5; HOMA-β = 20 × fasting serum insulin (mU/L) ÷ fasting plasma glucose (mg/dL)– 3.5. RD WT, Regular diet wild type; RD KO, Regular diet CCR2 knockout; HFD WT, High-fat diet wild type; HFD KO, High-fat diet CCR2 knockout.

*P < 0.05 compared to that in RD WT

#P < 0.05 compared to that in HFD WT.

### CCR2 knockout ameliorates lipid accumulation

**Fig 4:**
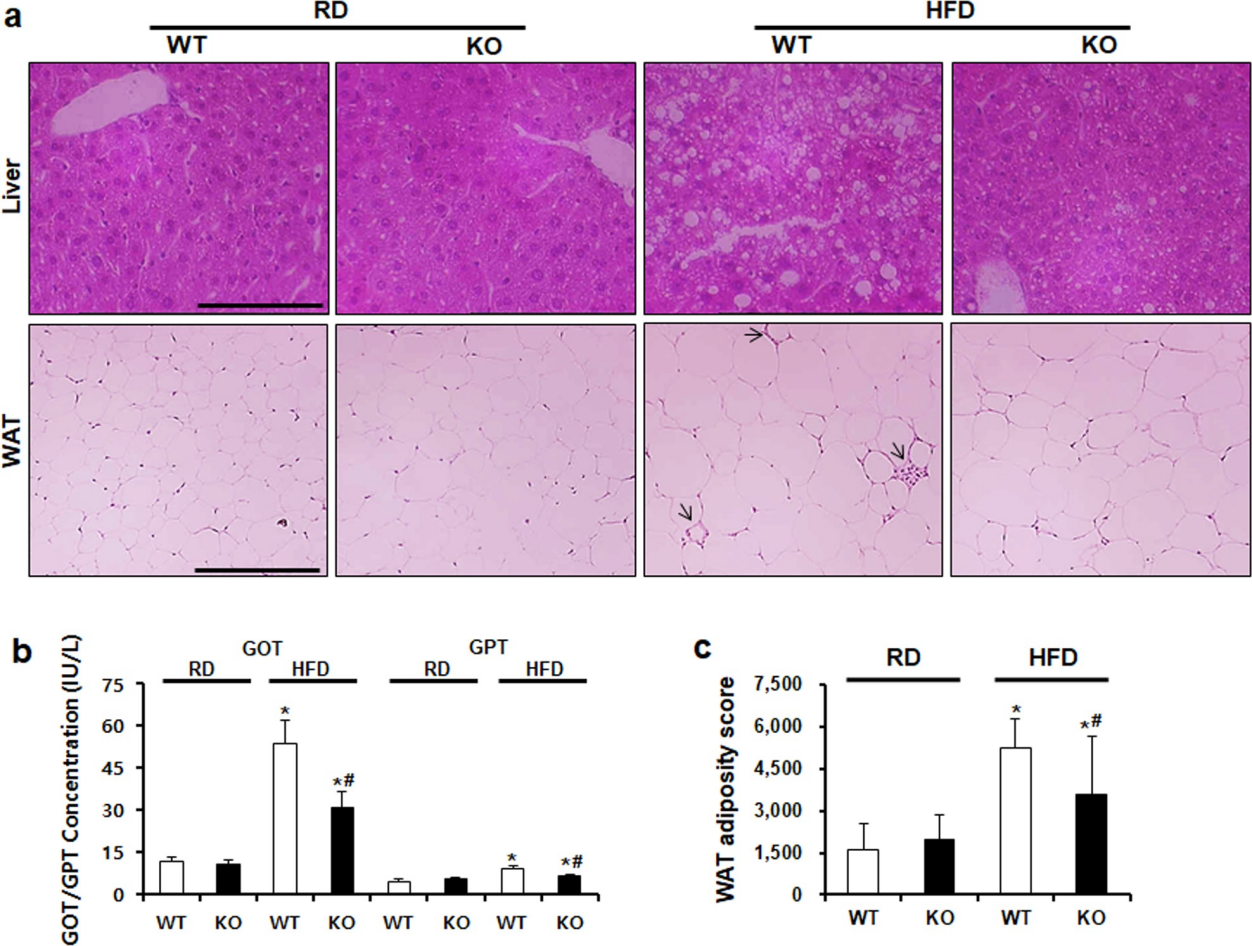
CCR2 depletion decreases lipid accumulation in the liver and WAT. (a) To analyze lipid accumulation in tissues, H&E staining was performed. Increased lipid droplets and fat size in HFD WT were decreased in HFD KO. Arrow indicates a crown-like structure. (b) GOT and GPT in mouse serum were measured using ELISA kit. Markedly increased concentration of GOT and GPT in HFD WT was significantly decreased in CCR2 KO. (c) Adiposity size of WAT was measured by Image J software. Adiposity size was significantly increased in HFD WT and markedly ameliorated by CCR2 KO similar to the result of WAT staining. Original magnification is ×100; bar = 50 μm. WAT, white adiposity tissue; RD WT, Regular diet wild type; RD KO, Regular diet CCR2 knockout; HFD WT, High-fat diet wild type; HFD KO, High-fat diet CCR2 knockout. *P < 0.05 compared to that in RD WT; #P < 0.05 compared to that in HFD WT.

Effect of CCR2 depletion on serum total cholesterol and triglycerides levels were investigated. Increased total cholesterol and triglycerides levels in HFD WT mice were significantly decreased in HFD KO mice (Fig 3C and 3D). The effect of CCR2 depletion on lipid accumulation in the liver and WAT was evaluated using H&E staining (Fig 4A). The liver in HFD WT mice showed increased lipid accumulation, while CCR2 depletion reduced fat accumulation in HFD KO mice. GOT and GPT liver damage markers were increased in HFD WT mice. However, in HFD KO mice, they were significantly recovered (Fig 4B). Regarding WAT, HFD feeding induced crown-like structure and increased the adiposity size. CCR2 depletion ameliorated HFD-induced injury in WAT of HFD KO mice (Fig 4A and 4C).

### CCR2 knockout ameliorates albuminuria and glomerular hypertrophy

**Fig 5:**
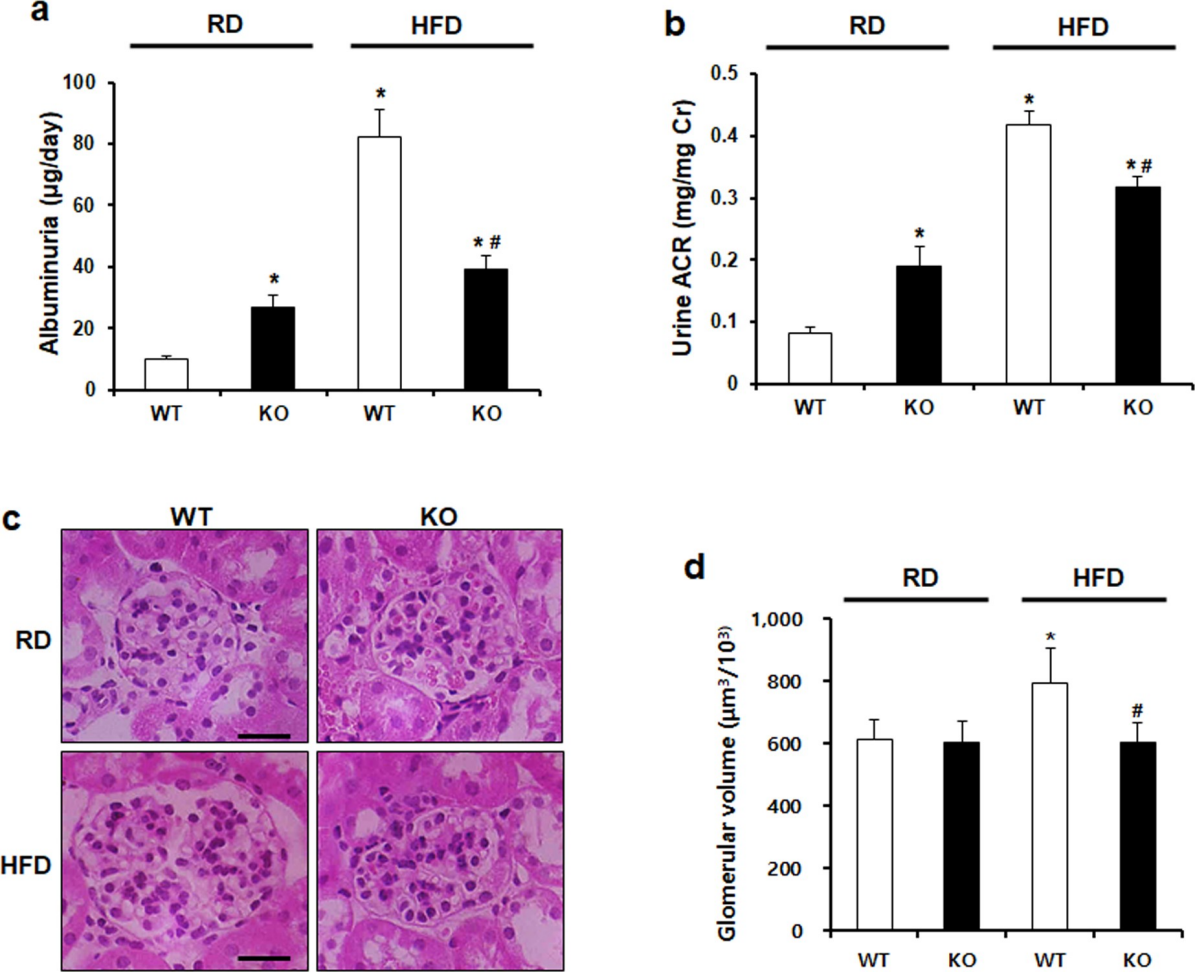
CCR2 depletion ameliorates HFD-induced albuminuria and glomerular hypertrophy. To examine kidney function, albuminuria and creatinine levels were measured by ELISA using 24-hour urine collection. (a, b) Elevated levels of albuminuria and urine albumin/creatinine ratio (ACR) in HFD WT were significantly improved by CCR2 depletion. (c) Representative images of H&E-stained kidney sections. (d) Glomerular volume was measured by Image analysis software. Increased glomerular volume in HFD WT mice was significantly restored by CCR2 depletion in HFD KO. Original magnification is x400; bar = 50 μm. RD WT, Regular diet wild type; RD KO, Regular diet CCR2 knockout; HFD WT, High-fat diet wild type; HFD KO, High-fat diet CCR2 knockout. *P < 0.05 compared to that in RD WT; #P < 0.05 compared to that in HFD WT.

To assess kidney function, we investigated albuminuria in 24-hours urine collection and obesity-induced glomerular volume. Significantly increased albuminuria and albumin to creatinine ratio (ACR) in HFD WT mice were restored in HFD KO mice (Fig 5A and 5B). HFD-induced increase in glomerular volume was also decreased by CCR2 depletion in HFD KO mice (Fig 5C and 5D).

### CCR2 knockout ameliorates podocyte injury and inflammation

**Fig 6:**
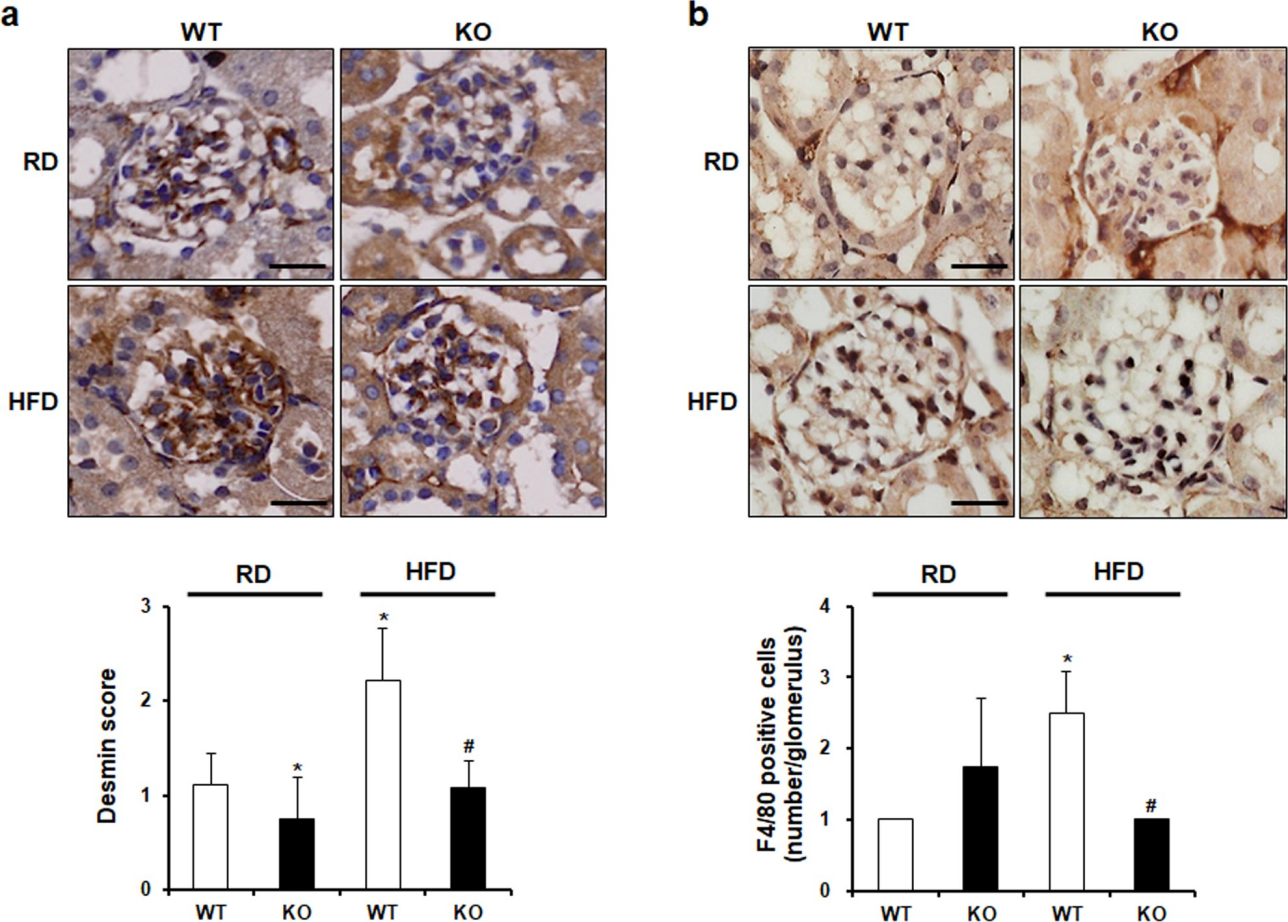
The effect of CCR2 depletion on podocyte injury and macrophage infiltration. (a) Desmin and (b) F4/80 staining were performed by immunohistochemistry. Desmin and F4/80 score were measured semiquantitatively (below). The level of staining was graded as follow: 0, 0%; 1+, 1–25%; 2+, 26–50%; 3+, 51%-75%; 4+, 76%-100%. Increased expression of desmin and F4/80 in HFD WT was significantly decreased in HFD KO mice. Original magnification is ×400; bar = 50 μm. RD WT, Regular diet wild type; RD KO, Regular diet CCR2 knockout; HFD WT, High-fat diet wild type; HFD KO, High-fat diet CCR2 knockout. *P < 0.05 compared to that in RD WT; #P < 0.05 compared to that in HFD WT.

Next, we performed desmin staining to examine the effect of CCR2 depletion on podocyte injury. In HFD WT mice, glomerular desmin expression was increased compared to that in RD WT. HFD-induced upregulation of desmin was reduced by CCR2 depletion in HFD KO mice (Fig 6A). To investigate the effect of CCR2 depletion on inflammation, proinflammatory cytokine TNF-α and macrophage marker F4/80 were checked. Increased macrophage infiltration in glomeruli of HFD WT mice was decreased by CCR2 depletion in HFD KO mice (Fig 5B). Similar to this, increased expression of TNF-α was also diminished in HFD KO mice (Fig 8B).

### CCR2 depletion improves both GBM thickness and podocyte shapes

**Fig 7:**
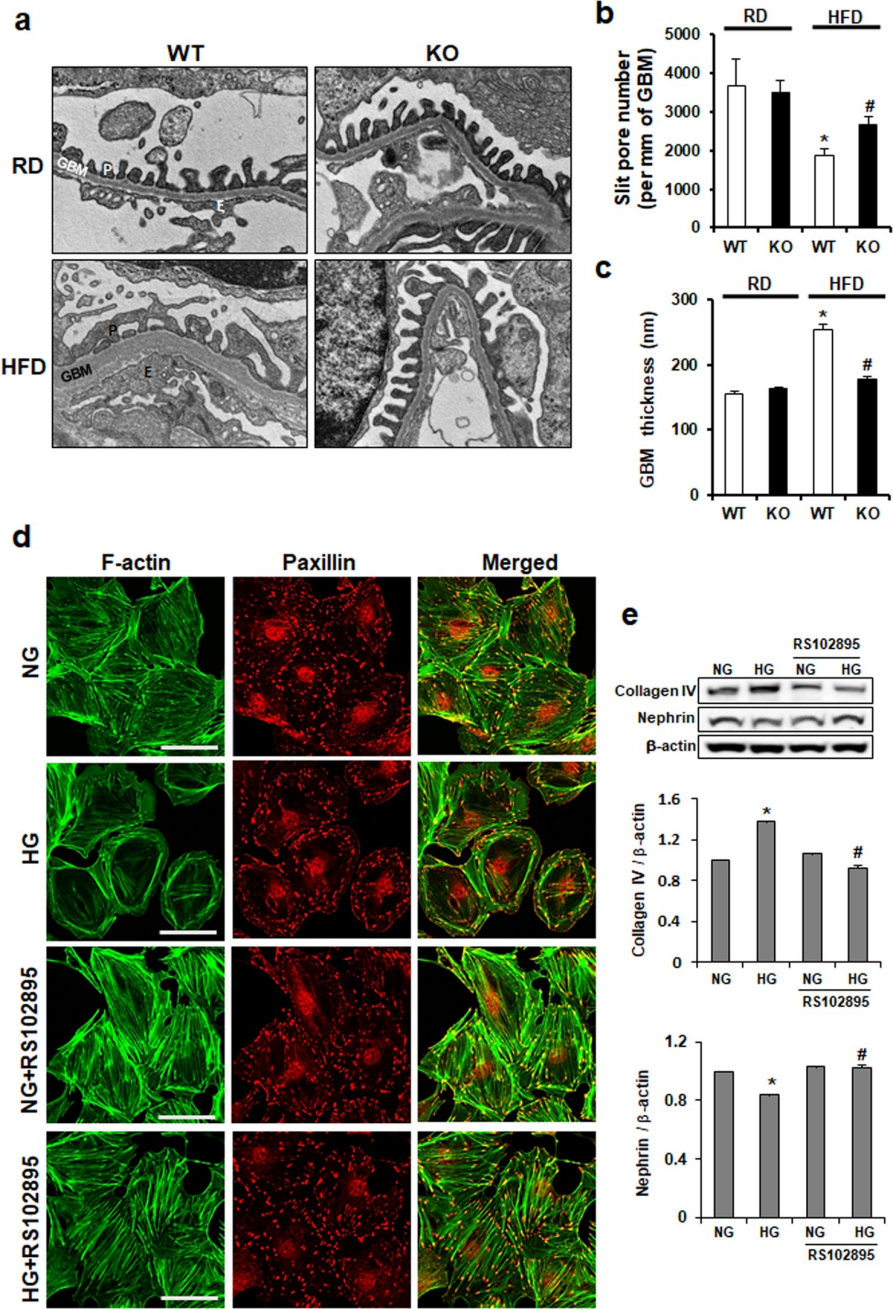
The effect of CCR2 depletion on glomerular structure. (a) Changes of glomerular structure were determined by EM. (b) The scores of slit pore number and (c) GBM thickness were measured using image analysis software. Abnormal glomerular structure in HFD WT was restored by CCR2 depletion in HFD KO. Original magnification is x30k. (d) Cultured podocytes grown on coverslips were fixed with 4% PFA and immunolabeled with FITC-phalloidin (green) and Paxillin (red). Treatment of podocytes with HG resulted in actin rearrangement, which was restored by CCR2 depletion. Magnification 40x; bar = 50 μm. (e) Western blots showing expression level of type IV collagen and nephrin in podocytes. P, Podocyte; GBM, Glomerular basement membrane; E, Endothelial cells; RD WT, Regular diet wild type; RD KO, Regular diet CCR2 knockout; HFD WT, High-fat diet wild type; HFD KO, High-fat diet CCR2 knockout. *P < 0.05 compared to that in RD WT; #P < 0.05 compared to that in HFD WT.

Changes of glomerular structure were investigated by EM. Although GBM and podocytes in RD groups were uniformly organized, GBM thickness and podocyte effacement were observed in HFD WT mice. However, those abnormal ultrastructures were improved by CCR2 depletion in HFD KO mice (Fig 7A). In addition, podocyte slit pore density and GBM thickness observed in HFD WT mice were restored in HFD KO mice (Fig 7B and 7C). To investigate whether those structural improvements observed in HFD KO kidney were by CCR2 depletion, we used immortalized mouse podocytes as *in vitro* system. Podocytes were treated by HG with or without CCR2 antagonist RS102895 and then podocytes were visualized by double staining with FITC-phalloidin dye and paxillin (Fig 7D). Uniformly organized actin stress fiber in normal podocytes was disorganized, decreased, and cortically rearranged by HG treatment. RS102895 restored the rearrangement of actin cytoskeleton. Furthermore, the increased expression of type IV collagen and the reduced expression of nephrin (slit diaphragm protein) by HG treatment were restored by CCR2 depletion (Fig 7E). These data suggest that CCR2 blockade could improve podocyte function and actin cytoskeleton.

### CCR2 knockout ameliorates ER stress and ROS in kidney

**Fig 8:**
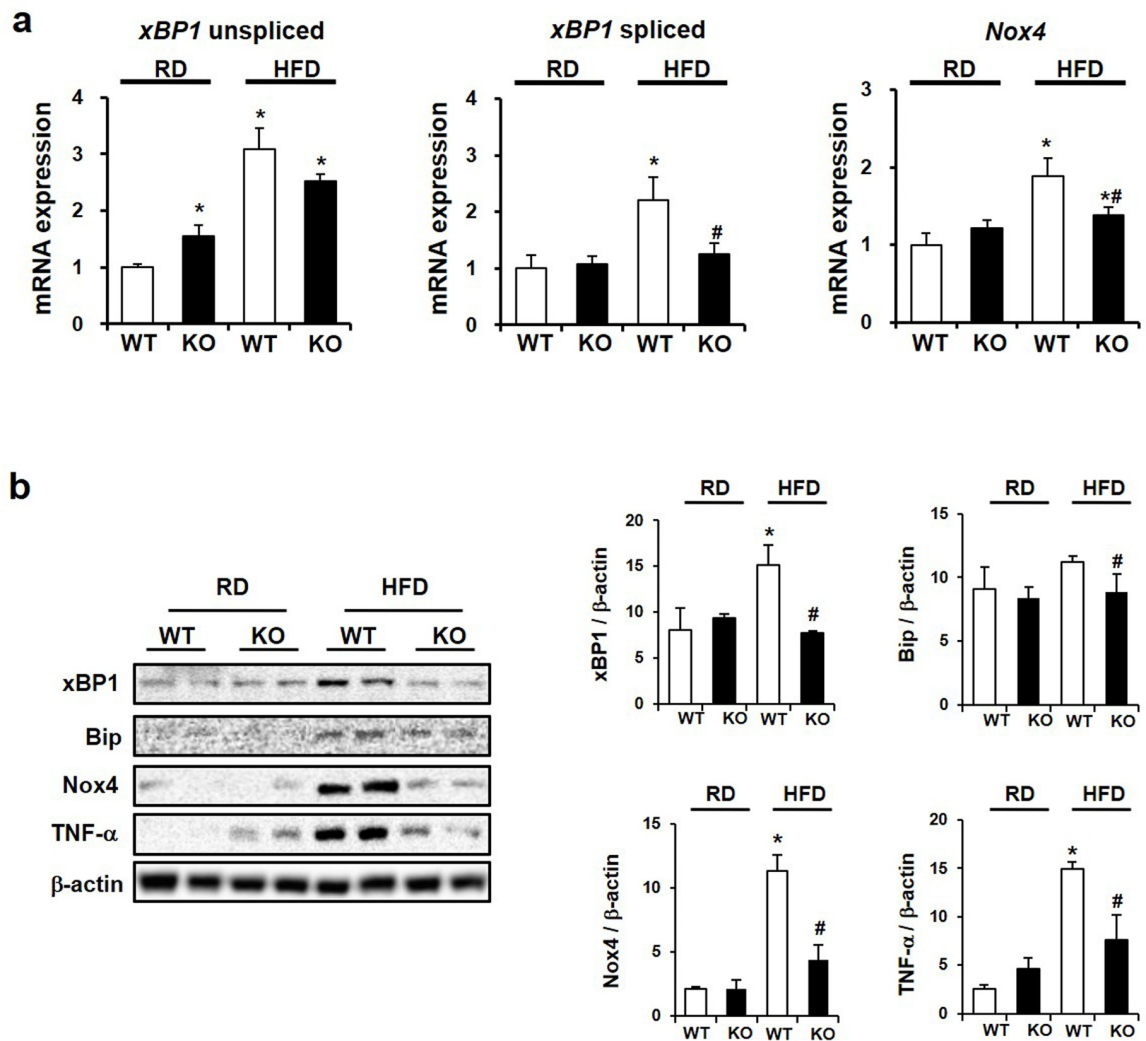
The effect of CCR2 knockout on ER stress and ROS. (a) mRNA levels of *xBP1* and *Nox4* by semiquantitative real-time PCR, corrected by *β-actin* mRNA level in the same sample. (b) Western blots (left) and densitometry (right) demonstrating that HFD-induced increases of xBP1, Bip, Nox4, and TNF-α were decreased by CCR2 depletion in HFD KO. Data are presented as the means ± SEM, *n* = 4 per group. RD WT, Regular diet wild type; RD KO, Regular diet CCR2 knockout; HFD WT, High-fat diet wild type; HFD KO, High-fat diet CCR2 knockout. *P < 0.05 compared to that in RD WT; #P < 0.05 compared to that in HFD WT.

Next, we investigated the changes of renal ER stress and ROS using semiquantitative real-time PCR and Western blots. Renal *xBP1* and *Nox4* mRNA expression were significantly increased in HFD WT mice. However, HFD-induced increases were significantly attenuated in HFD KO mice (Fig 8A). Consistent with these data, increased expression of xBP1, Bip, and Nox4 proteins in HFD WT mice was decreased by CCR2 depletion in HFD KO mice (Fig 8B).

## Discussion

In the recruitment of inflammatory cells to the kidney, chemokines play important roles. CCL2 is one chemokine that is mainly expressed in inflammatory cells after pro-inflammatory stimuli and tissue injury [35, 36]. It attracts macrophages by signaling through CCR2 chemokine receptors. It has been reported that CCL2 can induce the pathogenesis of diabetic glomerular sclerosis [37–40].

In the present study, we showed that HFD induced overt obesity characterized by increased body weight, fat accumulation, dyslipidemia, and hyperinsulinemia. We also found that HFD induced renal injury as evidenced by increased proteinuria, GBM thickness, glomerular hypertrophy, and abnormal ultrastructure of glomerular filtration barrier. However, CCR2 depletion restored these obesity-induced kidney injuries. In addition, it attenuated hyperinsulinemia and dyslipidemia.

In several obesity models, transcripts characteristic of macrophages is coordinately increased in direct proportion to body weight [41]. Increase macrophages number in the adipose tissue and the kidney is apparent in animal models of obesity [41]. We also demonstrated that macrophage infiltration and TNF-α expression were significantly increased by HFD feeding. However, CCR2 depletion significantly attenuated renal macrophage infiltration and TNF-α in HFD KO mice. These results suggested that CCR2 depletion ameliorated renal damage by restoring podocyte effacement, glomerular volume, and GBM thickness induced by HFD. Previously, our group has observed that RS102895, a CCR2 inhibitor, can restore diabetic-induced renal nephropathy and hepatic steatosis in *db/db* mice [42, 43]. Our previous study has also demonstrated that CCL2/CCR2 signaling axis might be a potential therapeutic target in diabetic kidney disease through improving podocyte permeability, actin cytoskeleton, and podocyte motility [44]. Tarabra *et al*. have demonstrated that CCL2 knockout could block the development of diabetic nephropathy and that CCL2 knockout is associated with the reduction of proteinuria and improvement of nephrin expression [45]. Consistent with these, HG-induced rearrangement of actin cytoskeleton and downregulation of nephrin in podocytes were restored by CCR2 inhibition, suggesting that CCR2 blocking could improve podocyte function. Therefore, in this study, we demonstrated the therapeutic effect of CCR2 depletion on nephropathy in a HFD-induced obese animal model.

Obesity has several effects on renal physiology, including insulin resistance, inflammation, oxidative stress, renin-angiotensin-aldosterone, and hyperlipidemia [6]. Our data showed that CCR2 depletion attenuated insulin resistance, hyperlipidemia, and fat accumulation induced by HFD, although CCR2 depletion did not significantly reduce body weight. Moreover, CCR2 depletion ameliorated renal injury through restoring fat accumulation, glomerular hypertrophy, and abnormal glomerular structure.

In obesity, elevated levels of fatty acids increase ROS production in the mitochondria [13, 14] and may impair the protein-folding machinery within ER as ER stress [46]. ER plays a principle role in controlling various cellular functions and fate. Because ER is very sensitive to alterations in cellular homeostasis, ER stress is induced by several pathological conditions [47, 48]. CCL2 expression can affect the production of reactive oxygen species, including ERK, Mn-SOD, HIF-1, and Nox [49]. ER/mitochondrial stress induces obesity-related high leptin levels [50]. ATF4 and xBP1 are ER stress-induced transcription factors. They are related to the production of CCL2, IL-6, IL-8, and CXCL3 by human aortic endothelial cells in normal state and in the presence of oxidized lipids [51]. In the present study, increased expression levels of Nox4, Bip, and xBP1 in the kidney indicating increased oxidative stress and ER stress were observed in HFD-induced obese mice. However, CCR2 depletion reduced Nox4, Bip, and xBP1 expressions.

In conclusion, our results suggest that blocking CCL2/CCR2 signaling pathway can ameliorate renal injury and proteinuria induced by HFD. These effects are at least produced by inhibiting oxidative stress and ER stress, thereby improving glomerular structure. Therefore, CCR2 depletion might be a novel therapeutic target against obesity-induced kidney injury.
